# Hello healthcare: evaluating the impact of a healthcare conference for secondary school pupils

**DOI:** 10.1186/s12909-025-07637-2

**Published:** 2025-07-31

**Authors:** Nadin Hawwash, Jacqueline Lavallee, Enam Haque

**Affiliations:** 1 School of Medical Sciences, Faculty of Biology, Medicine and Health, University of Manchester, Manchester, United Kingdom; 2 Cancer Research UK Manchester Cancer Research Centre, Manchester, United Kingdom

**Keywords:** Medicine, Healthcare, Widening participation, Widening access, Outreach

## Abstract

**Background:**

There are over 350 careers within healthcare. However, in the United Kingdom, opportunities are limited for secondary school pupils to learn about a variety of healthcare careers. We aimed to address this by delivering an in-person regional conference on healthcare careers for Year 10 pupils from widening participation (WP) backgrounds. This “Hello Healthcare Conference” enabled pupils to gain insight into a range of careers within healthcare. We explored the impact of the conference on pupils’ self-reported knowledge, skills, and attitudes to healthcare careers.

**Methods:**

We conducted a pre-test-post-test study of 44 pupils from WP backgrounds who attended Hello Healthcare to evaluate the effectiveness of the conference. A five-point Likert scale confidence questionnaire was used to evaluate the impact of Hello Healthcare on school pupils’ self-reported knowledge, skills, and attitude to healthcare careers. A Shapiro-Wilk test revealed a lack of normal distribution (*p* < 0.05); therefore, the pre-test-post-test results for each pupil were compared using a Wilcoxon signed-rank test.

**Results:**

44 Year 10 pupils from WP backgrounds across 4 schools were included in this study. Self-reported knowledge of most healthcare careers improved after the conference. Pupils’ perception of having the necessary skills for a healthcare career significantly increased, with Z = -5.78, *p* < 0.001, and large effect size (*r* = -0.87). Pupils’ perception that they could successfully apply for a healthcare course increased (Z = -5.52, *p* < 0.001, *r* =-0.83).

**Conclusions:**

The Hello Healthcare conference was beneficial in improving pupils’ awareness and attitudes towards healthcare careers and demonstrated an effective method by which to address the limited perceived knowledge WP pupils may have. Government and universities need to support and invest in the Hello Healthcare conference concept to replicate the impact of the intervention in other regions of the UK.

## Background

In the National Health Service (NHS), there are over 350 different NHS careers [1]. The Health Education England (HEE) (2014) strategy highlighted the importance of widening participation (WP) and developing a diverse workforce capable of responding to healthcare disparities, changes in population demographics, and subsequent healthcare demands [2]. WP focuses on increasing the participation of young people, particularly those from underrepresented groups (i.e. first-generation higher education pupils, pupils with low-income backgrounds and/or from schools with a low performance relative to the national average) [3]. Individuals from areas low in participation in higher education were less likely to undertake careers in healthcare [2]. WP initiatives established to address these findings include ‘Gateway’ or ‘Foundation’ courses that improve access to healthcare-related degrees. This is through the provision of ‘contextual offers,’ where grades are lowered for pupils from WP backgrounds who complete an outreach programme [2]. In 2021, the UK government stated that universities should focus on working with schools to increase aspirations and attainment and evidence this [4]. Although government and charity initiatives have supported some pupils, more work is necessary as, in the UK, access to healthcare degrees has become more difficult with the increased demand on admissions requirements which may further disadvantage WP pupils with limited insight into healthcare careers [5].

The Higher Education Access Tracker (HEAT) database is a longitudinal tracking database to identify activities most useful in higher education preparation [6]. We identified 503 UK schools located in WP postcode areas in the HEAT database by accessing the database and filtering out schools flagged with a WP postcode. The authors created the Plant a Seed series, an asynchronous online video resource to ‘Inspire,’ ‘Educate’ and Motivate Year 7–9 pupils to a career in Medicine [6]. However, this successful initiative would be difficult to replicate if covering a wide range of healthcare careers, as it requires a large commitment from schools, consisting of a 3-part video series for each healthcare career. Therefore, an alternative approach is required. The demonstrable impact of an in-person WP medicine conference by Ryan et al., (2021) on Year 12 pupils’ confidence [7], alongside findings by Martin et al. (2018), who identified the limited knowledge pupils in Year 9 had on the breadth of medical careers, led to the formation of the Hello Healthcare Conference [8].

“Hello Healthcare” is a novel initiative that provides efficient exposure to a variety of healthcare careers to improve the knowledge, skills and attitudes towards these careers so pupils from WP backgrounds do not have to rely on external exposures like family experiences in healthcare or networks etc. to make informed future decisions and early admissions preparations. We designed Hello Healthcare as an in-person conference with a focus on inspiring Year 10 pupils on healthcare by highlighting an array of healthcare-related degrees and careers. The conference consisted of workshops, talks, and a question-and-answer session to allow pupils to gain insight into the daily life of each healthcare professional, the impact and challenges involved in their work and the typical requirements needed to pursue each healthcare career. Year 10 pupils were specifically chosen to participate as they were at a pivotal stage in exploring career options, with most having selected relevant GCSE subjects for healthcare degrees, striving to achieve the required GCSE grades and undertaking the relevant work experience. We hypothesise early exposure to a variety of healthcare careers would allow more informed decision-making, early planning and preparation. Our study aimed to evaluate the impact of the conference on the self-reported knowledge, skills, and attitudes of pupils from WP backgrounds.

## Methods

### Participants

We included pupils in Year 10 from secondary schools in a WP-flagged location in Greater Manchester if they I) consented to this study ii) attended the Hello Healthcare Conference and iii) completed and handed in their pre- and post-conference feedback forms.

### Study recruitment

We recruited secondary schools through a collaborative network of local universities and schools that had high proportions of pupils from WP backgrounds and advertised the study using our university’s staff web pages. We distributed an initial study and conference advertisement to gauge interest and sent a detailed letter regarding the study and conference to schools that expressed interest. Teachers at the participating schools selected pupils to attend the series. We sent participant information sheets, consent forms and assent forms to each school. To minimise the administrative burden and associated costs, we also sent a stamped addressed envelope for the schools to return signed parental consent and assent forms. School participation required at least two members of staff from each school to be present at the conference. Staff were requested to support pupils at the conference by being present, chaperoning them through the different activities, and acting according to their school policy.

### Hello healthcare conference design

The Hello Healthcare conference took place in February 2024 and consisted of a student and doctor-led talk on their journey in healthcare; three workshops on different careers in healthcare, a networking session held by admissions teams and healthcare workers in the university faculty and a question and answers session held by student ambassadors (Table 1). We allocated pupils to one of two workshop “bundles” at random. Each workshop bundle consisted of three different healthcare career workshops. The admissions teams present at the networking sessions included Nursing, Allied Healthcare Professionals, Optometry, Life sciences, Dentistry, Medicine, and Physician Associates. Workshop facilitators were requested to include an introductory talk on their career and daily work, followed by an interactive workshop element that focuses on a skill primarily used in their practice and a 5-minute Q&A to end the workshop.

**Table 1:** Hello healthcare conference timetable

Time	Activity
09:30–10:00	Registration, pre-conference questionnaire
10:00–10:30:	Opening Lecture: A WP Student’s Journey.
10:40 − 11:30:	Workshop 1 (45 min) Q&A (5 min).
11:30–11:50	Break
11:50 − 12:40	Workshop 2 (45 min) Q&A (5 min).
12:50–13:40	Workshop 3 (45 min) Q&A (5 min).
13:40– 14:30	Lunch
14:30– 15:20	Networking with professionals, stalls, and admissions team
15:00–16:00	Closing Lecture: A WP GP’s Journey, student ambassador Q&A session and post-conference questionnaire.

Students were pre-allocated to one of the workshop bundles below

Workshop Bundle 1: Nursing workshop, Dentistry workshop, Physician associate workshop

Workshop Bundle 2: Optometry workshop, Medicine workshop, Pharmacy workshop

Pupils were provided with a welcome pack containing a pen, notepad, name lanyard and conference booklet. The booklet included conference details and short interviews with healthcare professionals, exploring their roles and motivations, as well as highlighting career opportunities and admissions information. Professionals included in the booklet were a general practitioner, consultant surgeon, professor in Health Informatics, pharmacy lecturer, dentist, audiologist, physician associate student, optometrist, and a nurse.

### Study design

We provided pupils with pre- and post-conference questionnaires in their welcome packs on arrival to the conference (Table 2). We asked them to complete the pre-conference questionnaire at the start of the welcoming lecture. Pupils then attended the conference sessions and were asked to complete the post-conference questionnaire at the end of the closing lecture (Table 1).

**Table 2:** Hello healthcare conference questionnaire

	Strongly disagree	Disagree	Undecided	Agree	Strongly agree
**Knowledge**					
I am aware of what the following healthcare workers do					
Nurse					
Optometrist					
Doctor					
Pharmacist					
Physician associate					
Paramedic					
Dentist					
Data scientist					
Speech and language therapist					
Surgeon					
**University**					
I know what the NHS is					
I understand the different careers in the NHS					
I know the steps needed to study for a healthcare career					
**Skills**					
I feel I have the skills needed to work in healthcare					
**Attitude**					
I feel able to successfully apply for a healthcare course					
I feel I have the qualities needed to work in healthcare					

### Questionnaire

We used the same questionnaire for both the pre-and post-conference data collection and ensured that each pupil had the same specific number for both questionnaires. Numbered questionnaires were included in the welcome packs and the packs were given to each pupil. The questionnaire consisted of 16 questions focused on the themes: of self-reported knowledge, skills and attitudes and had a five-point Likert scale from “Strongly Agree” to “Strongly Disagree” (Table 2). Quantitative data was only collected, given the nature of the conference, and time demands in completing the questionnaire. The authors did not collect the characteristics of each pupil, so data collection was anonymous.

### Statistical analysis

The distribution of the data was first assessed with a Shapiro-Wilk test which found a lack of normal distribution of the data (*p* < 0.05). Consequently, the Wilcoxon signed-rank test was used to statistically compare pre- and post-conference questionnaire results to identify whether the Hello Healthcare conference had an impact on pupils’ self-reported knowledge, skills, and attitudes to healthcare in line with Bandura’s Social Cognitive Theory, which emphasises the dynamic interaction of personal, environmental and behavioural factors [9].

## Results

47 pupils from 4 secondary schools in Greater Manchester, participated in the Hello Healthcare Conference and 44 pupils were included in this study. 3 pupils were excluded as their questionnaire was not handed in at the end of the conference.

### Knowledge

**Fig. 1:**
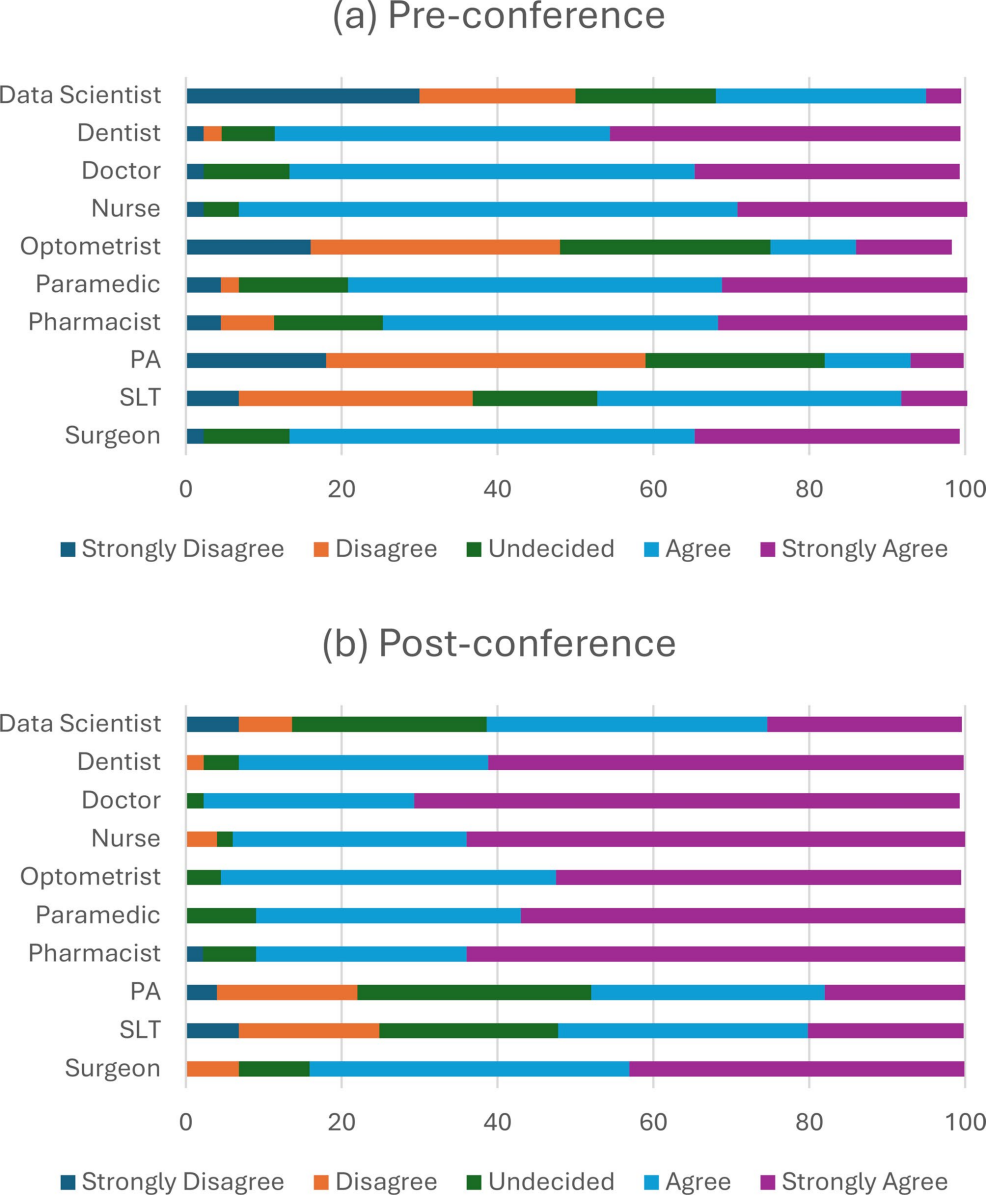
Pupils’ perception of their knowledge of different healthcare specialities (**a**) pre-conference and (**b**) post-conference. Abbreviations: SLT, speech and language therapy, PA, physician associate

After completing the Hello Healthcare conference, pupils had a significant increase (*p* < 0.05) in their self-reported knowledge of the role of a nurse, doctor, pharmacist, optometrist, paramedic, and dentist (Table 3). Knowledge of data science and physician associate roles was increased by a median of 1 unit on the Likert scale when comparing pre- and post-conference results (*p* < 0.001) suggesting an overall improvement in their perceived knowledge of these subjects. Pupils’ self-reported knowledge of the role of a speech and language therapist and surgeon did not improve after the intervention (Fig. 1; Table 3). 24.8% of pupils disagreed or strongly disagreed that the conference improved their perceived knowledge of speech and language therapy, and 6.8% disagreed that the conference improved their perceived knowledge of surgery as a career.

**Table 3:** Comparison of pre-and post-conference perceived knowledge of healthcare careers

Healthcare career	Z-score	*p*-value	Effect size, *r*
Data Scientist	-5.05	0.00014	-0.76
Dentist	-5.02	0.01892	-0.74
Doctor	-5.59	0.00050	-0.84
Nurse	-5.24	0.01836	-0.79
Optometrist	-5.33	0.00000	-0.80
Paramedic	-5.40	0.00255	-0.81
Pharmacist	-5.08	0.00248	-0.77
PA	-4.75	0.00046	-0.72
SLT	-3.40	0.25602	-0.51
Surgeon	-4.75	0.78533	-0.72

Abbreviations: SLT, Speech and Language Therapy, PA, Physician Associate

NB: Negative Z-score and effect size, r values indicate an improvement from pre- to post-conference scores

**Fig. 2:**
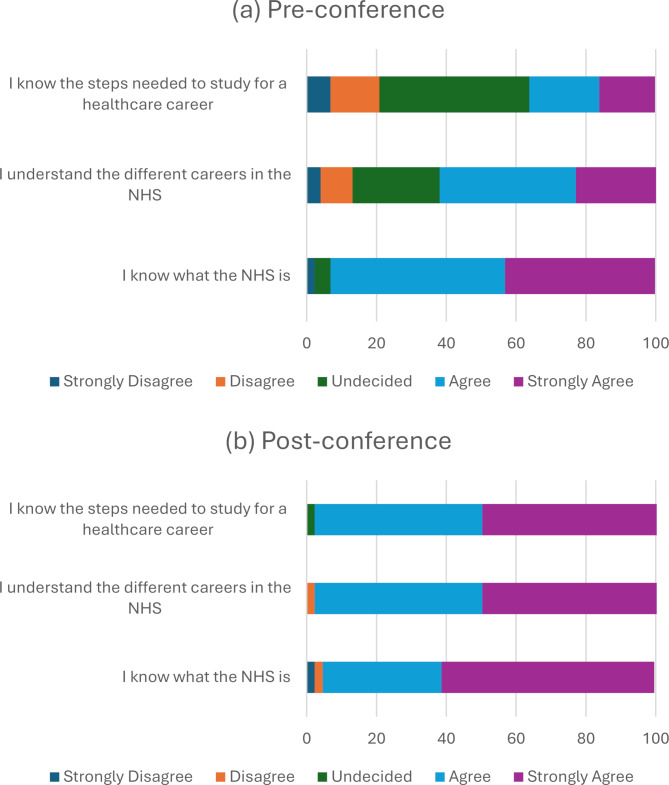
Pupils’ perception of their knowledge of the national health service and healthcare careers (**a**) pre-conference and (**b**) post-conference. Abbreviations: NHS, national health service

Pupils’ perceptions of their knowledge of the NHS did not significantly change after attending the Hello Healthcare conference (Z = -5.38, *p* = 0.12352, *r* = -0.81), with an increase from 73% before to 95% after the conference. The conference significantly increased pupils’ understanding of different careers (Z = -5.65, *p* < 0.001, *r* = -0.85) (Fig. 2). Additionally, pupils’ knowledge of the steps required to study for a healthcare career increased with a median increase of 1 unit on the Likert scale (Z = -5.48, *p* < 0.001, *r* = -0.83).

### Skills

**Fig. 3:**
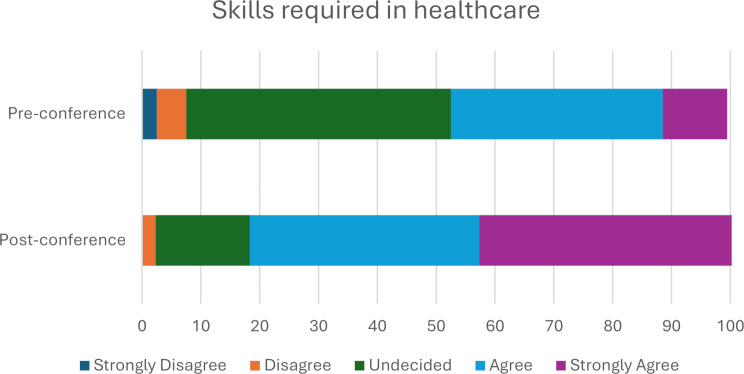
Pupil’s perception of having the skills required in healthcare

Pre-conference, 45% of pupils were undecided on whether they perceived to have the necessary skills to work in healthcare. Pupils reported a significant increase in their perceived skills required in healthcare following completion of the Hello Healthcare conference with a (Z = -5.78, *p* < 0.001), with a large effect size (*r* = -0.87) and a median increase of 1 unit on the Likert scale demonstrating an overall improvement in self-reported skills on attending the Hello healthcare conference (Fig. 3).

### Attitude

**Fig. 4:**
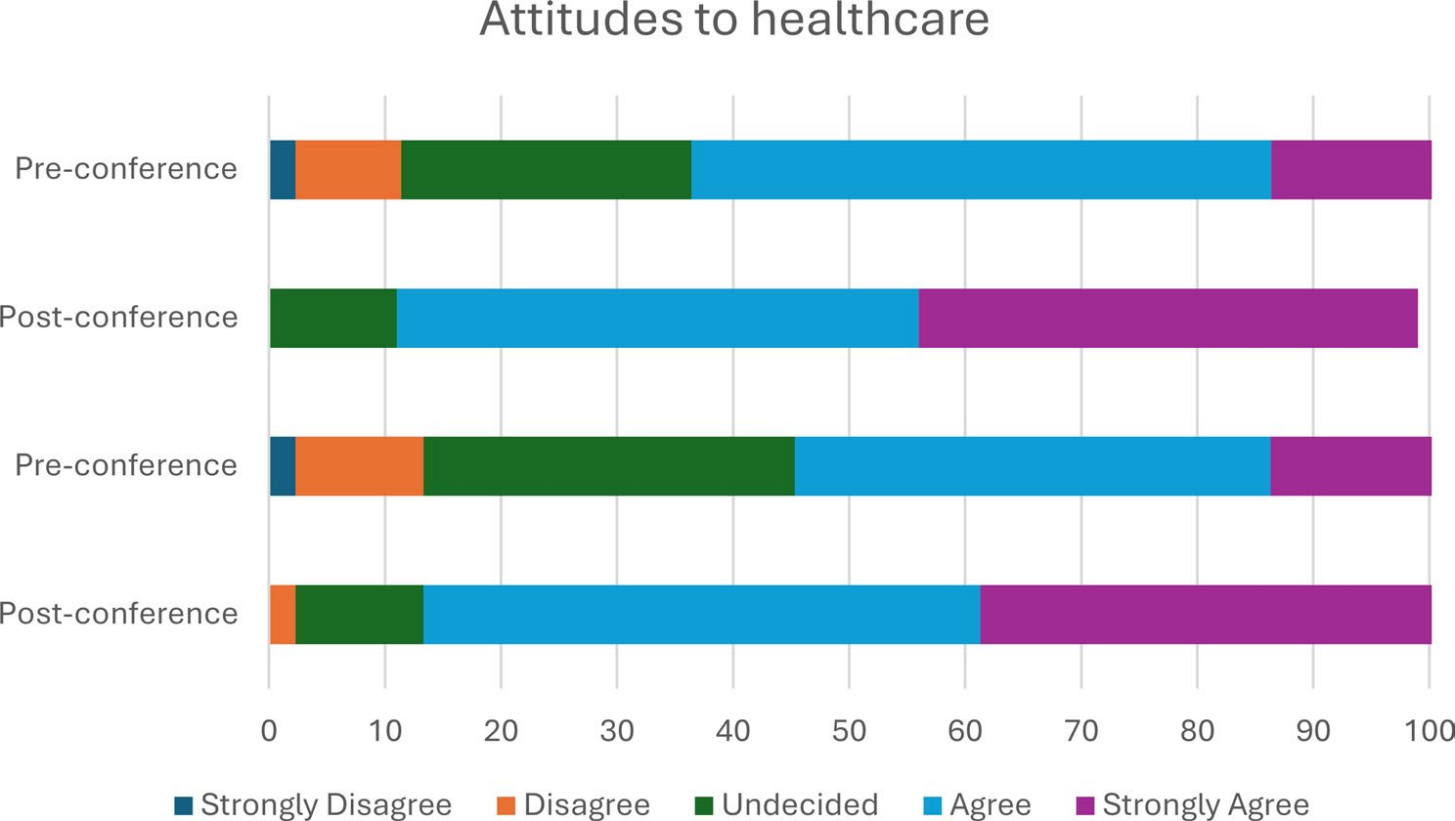
Pupil’s attitudes towards healthcare

Pre-conference only 14% of pupils strongly agreed to feeling able to successfully apply for a healthcare course and 32% were undecided; compared with the post-conference 39% of pupils strongly agreed to feeling able to apply for a healthcare course with 11% remaining undecided (Fig. 4). Overall, there was an increase in pupils’ perception that they could successfully apply for a healthcare course after attending Hello Healthcare (Z = -5.52, *p* < 0.001, *r* = -0.83) with a median increase of 1 unit on the Likert scale indicating an overall improvement in pupils self-reported attitudes to applying to a healthcare course as a result of attending the Hello Healthcare conference (Fig. 4).

## Discussion

Our study demonstrates the positive impact an in-person healthcare-focused conference can have on pupils’ self-reported knowledge, skills, and attitudes to healthcare. Although current findings are limited in generalisability to the pupil group analysed, given the small sample size, there is demonstrable potential for the beneficial impact of Hello Healthcare at scale.

There was an evident increase in pupils’ self-reported knowledge in all healthcare subjects that had an allocated 45-minute workshop (nursing, dentistry, medicine, pharmacy, optometry, and physician associate). Knowledge also increased in data scientist and paramedic roles. These careers were only showcased in the welcome talk, conference booklet and networking session. There was no significant increase in pupils’ self-reported knowledge of surgery and speech and language therapy. This may potentially be due to the lack of a specific workshop in the conference workshop design, where pupils could spend more time experiencing an interactive element related to those careers. However, this is on the assumption a workshop is more effective in improving pupils’ self-reported knowledge than the networking and booklet. Students primarily had some prior knowledge of these roles before the conference, so the limited increase in perceived knowledge may be attributed to their already sufficient self-reported understanding of these careers. Pupils reported an increased perceived understanding of the steps and skills required to study for a healthcare career demonstrating the beneficial impact of the conference. Exposure to hands-on practical skills within the workshops improved pupils’ perceptions of qualities needed to work in healthcare and how to successfully apply to a healthcare course.

Understanding the NHS is pivotal for a healthcare career in the UK. Pupils reported trivial improvement in perceived understanding of the NHS after the conference potentially due to the sufficient exposure of the NHS in the school curriculum and media. It could also be due to the lack of an NHS-specific workshop.

Pupils reported an increased understanding of healthcare careers and the steps and skills required to study for them, which was the primary purpose of the conference. The improved perceived knowledge and exposure to hands-on practical skills within workshops specific to each healthcare career improved pupils’ perceptions that they could successfully apply for a healthcare course, and they possess the necessary qualities. This demonstrated the short-term improvement a one-day conference had on the group of pupils analysed; thus, the conference should be repeated at scale to enhance the generalisability of findings.

Overall, the literature on initiatives that raise awareness of numerous healthcare careers to students from WP backgrounds is limited. The limited exposure to healthcare careers was, however, noted by Dutta et al. (2022), who formulated a Widening Access to Careers in Community Healthcare (WATCCH) programme at Imperial College London to raise 16-18-year-old pupils’ awareness of healthcare careers [10]. The programme covered medicine, nursing, physiotherapy, physician associate and pharmacy and arranged a series of monthly structured educational workshops, work experience and mentoring from undergraduate medical students [10]. They found improvements in students’ exposure to different healthcare careers following the completion of the programme but noted a key limitation that only doctors were involved in the design and delivery of the activities. We overcame this in Hello Healthcare by developing the workshops through collaboration with different healthcare professionals. However, the challenge was the reliance on the availability of colleagues. The WATCCH programme required a greater time commitment than Hello Healthcare, but both achieved similar outcomes of increasing awareness of healthcare careers.

It is crucial to acknowledge that adopting the Hello Healthcare Conference concept nationally may be a challenge due to the variation among universities as to their level of commitment to WP. A study by Cleland et al. in 2014 found that institutions “that aspired to be elite” were more likely to superficially map WP practices [11]. They also noted that analysis of the impact of WP initiatives was often lost, thus demonstrating the importance of the approach in our study.

### Strengths

This is the first study of a novel regional in-person conference formed through the collaboration of healthcare-related specialities/degrees. Only 3 participants were excluded due to a lack of questionnaire completion, so most conference attendees were analysed, which is a study strength; however, a potential response bias may have occurred, as more engaged pupils may have completed or more accurately completed the questionnaire. To mitigate this, an allocated period in the conference was provided for all attendees to complete the pre-and post-conference questionnaire. Participant selection bias was mitigated by the organisers providing all pupils with the necessary learning resources, as well as providing free breakfast and lunch. The conference was held during school hours to minimise selection bias of WP pupils with out-of-hours work commitments and/or difficulties in arranging independent conference travel.

### Limitations

The Hello Healthcare conference was scheduled to take place in May 2023. However, the ethics review process was delayed, giving schools limited time to arrange for the pupil and parent consent forms to be disseminated, completed, and returned. Consequently, the conference had to be postponed to July 2023 which was unfortunately also impacted due to the national teachers’ strike taking place on the same day as the conference. Therefore, the conference was scheduled for February 2024. Rescheduling the conference on two occasions may have contributed to the small number of participating schools as well as fewer healthcare careers being covered in the conference. The limited sample size may limit the generalisability of the findings of this study. Additionally, a potential selection bias may have been involved with more motivated schools participating in the conference.

There was a capacity for 120 pupils at the Hello Healthcare conference. However, recruitment was challenging, with schools dropping out due to time clashes with exams, work experience week, and staff absences. Further selection bias may have occurred with more motivated and well-resourced schools with capacity for attendance of two staff members may have only participated. For safeguarding, health and safety and school policy reasons, a minimum of 2 members of school staff for 15 pupils was required. Other factors included schools being concerned about pupils missing a full day of classes and teachers and teachers would miss teaching to support the pupils. We selected a weekday conference, to enable pupils to attend with their school and avoid travel payments. Schools were liable for travel expenses, which may have reduced uptake. A further limitation was the administrative burden, with the requirement to return pupil and parent consent forms. We attempted to overcome this by keeping paperwork to a minimum where possible and providing a pre-paid envelope to schools to avoid the need to scan signed forms. Another limitation is that demographic data could not be collected for anonymity limiting the interpretability of how representative the sample of pupils analysed was.

Kirkpatrick’s framework is a method for assessing how well outcomes are achieved [12]. The “Hello Healthcare” Conference evaluation focused on the first two parts of this model, examining pupil satisfaction and addressing improved knowledge, skills, and attitudes. However, the conference did not explore outcomes such as behavioural change or longer-term impacts, which are higher targets in Kirkpatrick’s model. These outcomes may be considered for future expansion of the study.

### Future work

 This study evaluated if the Hello Healthcare conference increased pupils’ understanding of various healthcare careers and related degrees. Analysis at scale is required across different regions to truly identify its impact. We also suggest a review of the efficacy of various approaches to conducting Hello Healthcare, to understand how to maximise impact and exposure to healthcare careers. Although there was a networking session highlighting a variety of healthcare careers, each pupil could only attend three healthcare workshops. Therefore, future work could evaluate whether it is more effective to keep workshop time to a minimum while maximising the number of workshops pupils attend. To implement the programme at scale, a standardised framework for the healthcare workshops is required. To do so would require an evaluation of individual workshops. Enrolment at scale would require identification of ways to minimise the administrative burden of Hello Healthcare, as this may have impacted school participation and thus conference attendance. Future work could also compare whether a one-off event such as Hello Healthcare versus a longer-term approach such as that adopted by the WATCCH study is more effective in improving pupils’ awareness on a variety of healthcare careers.

## Conclusion

Our study demonstrates that the Hello Healthcare conference increased the knowledge, skills, and attitudes to healthcare careers of pupils from disadvantaged backgrounds and thus will be adopted as an initiative at a northern university. To build a sustainable initiative, we invite government and academic institutions to collaborate with us in supporting the funding and maintenance of Hello Healthcare.

## Data Availability

The datasets used and/or analysed during the current study are available from the corresponding author upon reasonable request.

## Abbreviations

WP: Widening participation
